# On the Berry-Esséen bound of frequency polygons for *ϕ*-mixing samples

**DOI:** 10.1186/s13660-017-1336-9

**Published:** 2017-03-23

**Authors:** Gan-ji Huang, Guodong Xing

**Affiliations:** 1 College of Mathematics and Information Science, Guangxi University, Nanning, Guangxi China; 2 School of Mathematical Sciences, Xiamen University, Xiamen, Fujian China

**Keywords:** 60G05, 62G20, Berry-Esséen bound, uniformly asymptotic normality, *ϕ*-mixing, frequency polygons

## Abstract

Under some mild assumptions, the Berry-Esséen bound of frequency polygons for *ϕ*-mixing samples is presented. By the bound derived, we obtain the corresponding convergence rate of uniformly asymptotic normality, which is nearly $O(n^{-1/6})$ under the given conditions.

## Introduction

At first, we introduce briefly the conception of *ϕ*-mixing sequence. Set

1.1 $$ \phi(n) =\sup_{k\geq1} \sup_{A\in{\mathcal{{F}}}_{1}^{k}, B\in{\mathcal{{F}}}_{k+n}^{\infty }, P(A)>0} \bigl\vert P(B)-P\bigl(B\vert A\bigr)\bigr\vert , $$

where $\mathcal{{F}}_{1}^{k}=\sigma(X_{j}, 1\leq j\leq k)$ and $\mathcal{{F}}_{k+n}^{\infty}=\sigma(X_{j}, j> k+n)$. The sequence $\{X_{i}, i\geq1\}$ is called *ϕ*-mixing if $\lim_{n\to\infty} \phi(n)=0$. The *ϕ*-mixing dependence was introduced by Dobrushin [1], and many applications have been found. See, for example, Dobrushin [1], Utev [2], Yang [3], Yang and Hu [4] and so on.

In what follows, let us introduce the conception of frequency polygon. Suppose that *X* is a random variable with a density function $f(x)$, and let $X_{1}, X_{2}, \ldots, X_{n}$ be the sample drawn from the population *X*. Consider a partition $\cdots< x_{-2}< x_{-1}< x_{0}< x _{1}< x_{2}<\cdots$ of the real line into equal intervals $I_{k}=[(k-1)b _{n}, kb_{n})$ of length $b_{n}$, where $b_{n}$ is the bin width. For given $x\in R$, there exists $k_{0}$ such that $(k_{0}-\frac{1}{2})b _{n}\leq x<(k_{0}+\frac{1}{2})b_{n}$. Consider the two adjacent histogram bins $I_{k_{0}}=[(k_{0}-1)b_{n},k_{0}b_{n})$ and $I_{k_{1}}=[k _{0}b_{n},k_{1}b_{n})$, where $k_{1}=k_{0}+1$. Define $v_{k_{0}}=\sum^{n}_{i=1}I((k_{0}-1)b_{n}\leq X_{i}< k_{0}b_{n})$ and $v_{k_{1}}=\sum^{n}_{i=1}I(k_{0}b_{n}\leq X_{i}< k_{1}b_{n})$, which are the number of observations falling in the intervals mentioned above, respectively. The values of the histogram in these previous bins can be denoted by $f_{k_{0}}=v_{k_{0}}n^{-1}b_{n}^{-1}$ and $f_{k_{1}}=v_{k_{1}}n^{-1}b _{n}^{-1}$. Then the frequency polygon $\widehat{f}(x)$ can be defined as

1.2 $$ \widehat{f}(x)= \biggl( \frac{1}{2}+k_{0}-\frac{x}{b_{n}} \biggr) f_{k_{0}} + \biggl( \frac{1}{2}-k_{0}+ \frac{x}{b_{n}} \biggr) f_{k_{1}} $$

for $x\in[ ( k_{0}-\frac{1}{2} ) b_{n}, ( k_{0}+ \frac{1}{2} ) b_{n})$.

As pointed out by Scott [5], the frequency polygon has convergence rates similar to those of kernel density estimators and greater than the rate for a histogram. As for computation, the computational effort of the frequency polygon is equivalent to the one of the histogram. For large bivariate data sets, the computational simplicity of the frequency polygon and the ease of determining exact equiprobable contours may outweigh the increased accuracy of a kernel density estimator. Bivariate contour plots based on millions of observations are increasingly required in applications including high-energy physics simulation experiments, cell sorters and geographical data representation. Moreover, such data are usually collected in a binned form. Therefore, the frequency polygon can be a useful tool for examination and presentation of data. Since the frequency polygon has the advantages mentioned above, it attracts the attention of some scholars, and they have derived some results. For the explicit results obtained, one can refer to the references listed in Yang and Liang [6] and Xing et al. [7], which gave the strong consistency of frequency polygons. Among the obtained results, the study on asymptotic normality can be found in Carbon et al. [8]. The relevant Berry-Esséen bound for *ϕ*-mixing samples has not been seen. This motivates us to investigate the Berry-Esséen bound of frequency polygon under *ϕ*-mixing samples. Under the given assumptions, we give the corresponding Berry-Esséen bound. Furthermore, by the obtained Berry-Esséen bound, the relevant convergence rate of uniformly asymptotic normality is also derived, which is nearly $O(n^{-1/6})$ under the given conditions.

Throughout this article, we always suppose that *C* denotes a positive constant which only depends on some given numbers and may vary from one place to another. The organization of this paper is as follows. Section 2 contains the main result obtained, Section 3 contains the corresponding proof.

## Main result

For the convenience of formulation of our main result, we need the following assumptions.

(A1): The density function $f(x)$ is continuous in $x\in R$ and $f(x)\leq M$ for $x\in R$ and some $M>0$.
(A2): The random sample $\{X_{i}, 1\leq i\leq n\}$ is stationary, identically distributed and *ϕ*-mixing with $\phi(n)=O(n^{- \rho})$, where $\rho>\frac{7-58\epsilon}{36\epsilon}$ with $0<\epsilon<\frac{57}{1650}$.
(A3): The bin width $b_{n}$ satisfies $b_{n}\rightarrow0$ and $nb_{n}\rightarrow\infty$.
(A4): Define $\sigma^{2}_{n}(x):=\operatorname{Var}(\widehat{f}(x))$, and there exist $\delta>0$, two positive integers $p:=p_{n}$ and $q:=q_{n}$ such that
  $$p+q\leq n,\qquad qp^{-1}\leq C< \infty $$
  and
  $$\gamma_{1n}\rightarrow0,\qquad \gamma_{2n}\rightarrow0,\qquad \gamma_{3n} \rightarrow0, \qquad u_{1}(q)\rightarrow0,\qquad u_{2}(q)\rightarrow0, $$
  as $n\rightarrow\infty$, where
  $$\begin{aligned}& \gamma_{1n}:=q(p+q)^{-1}\bigl(nb_{n} \sigma^{2}_{n}(x)\bigr)^{-1},\qquad \gamma_{2n}:=p \bigl(nb _{n}^{1/2}\sigma_{n}(x)\bigr)^{-2}, \\& \gamma_{3n}:=p^{\delta/2}(nb_{n})^{-(1+\delta)}\bigl( \sigma_{n}(x)\bigr)^{-(2+ \delta)},\qquad u_{1}(q):=q^{-\rho/2+1} \bigl(nb_{n}^{3/2}\sigma_{n}^{2}(x) \bigr)^{-1} \end{aligned}$$
  and
  $$u_{2}(q):=\bigl(q^{\rho}pb_{n}\sigma_{n}^{2}(x) \bigr)^{-1/2}. $$

Based on the above assumptions, our main result can be given as follows.

### Theorem 2.1

*Suppose that assumptions* (A1)-(A4) *are satisfied*. *Then we have*

2.1 $$ \sup_{u}\bigl\vert F_{n}(u)-\Phi(u)\bigr\vert \leq C\bigl\{ \gamma _{1n}^{1/3}+\gamma _{2n}^{1/3}+ \gamma_{3n}+u_{1}(q)+u_{2}^{1/2}(q)\bigr\} , $$

*where* $F_{n}(u)=P(S_{n}< u)$, $S_{n}=\sigma_{n}(x)^{-1}\{\widehat{f}(x)-E \widehat{f}(x)\}$ *and* $\Phi(u)$ *is the distribution function of the standard normal random variable*.

By Theorem 2.1, the following corollary can be obtained directly.

### Corollary 2.1

*Let the conditions of Theorem* 2.1 *be satisfied*, *and let* $p=[n^{\tau}]$, $q=[n^{2\tau-1}]$, $\delta=10/9$, $b_{n}=O(n^{-1/5})$ *and* $\sigma_{n}(x)=O(n^{-2/5})$ *in* (2.1), *where* $\tau=1/2+3\epsilon$. *Then*

2.2 $$ \sup_{x\in R}\bigl\vert \widehat{f}(x)-f(x)\bigr\vert =O \bigl(n^{-1/6+5\epsilon/3}\bigr). $$

### Remark 2.1

By (2.2), the convergence rate is nearly $O(n^{-1/6})$ if $\epsilon\rightarrow0^{+}$, as desired. Correspondingly, Yang and Hu [4] gave also the Berry-Esséen bounds of kernel density estimator under *ϕ*-mixing samples and obtained the relevant rate of convergence $O(n^{-1/6}\log n \log \log n)$. Obviously, the two convergence rates are similar.

## Proof

Before proving Theorem 2.1, we give some denotations used later. Let

3.1 $$\begin{aligned}& Y_{i,k_{s}}=I\bigl\{ (k_{s}-1)b_{n}\leq X_{i}< k_{s}b_{n}\bigr\} ,\quad s=0,1, \end{aligned}$$

3.2 $$\begin{aligned}& e_{i,k_{s}}(x):=Y_{i,k_{s}}(x)-EY_{i,k_{s}}(x),\quad i=1,2,\ldots,n, s=0,1, \end{aligned}$$

3.3 $$\begin{aligned}& \eta_{i}(x):= \biggl[ \biggl(\frac{1}{2}+k_{0}- \frac{x}{b_{n}}\biggr)e_{i,k_{0}}(x) +\biggl( \frac{1}{2}-k_{0}+ \frac{x}{b_{n}}\biggr)e_{i,k_{1}}(x) \biggr] , \end{aligned}$$

3.4 $$\begin{aligned}& Z_{n,i}:=\bigl(nb_{n}\sigma_{n}(x) \bigr)^{-1}\eta_{i}(x), \end{aligned}$$

and let *p*, *q* as in assumption (A4), $v:=v_{n}=[n/(p+q)]$,

$$\begin{aligned}& l_{m,p,1}=(m-1) (p+q)+1, \qquad l_{m,p,p}=(m-1) (p+q)+p, \\& l_{m,q,1}=(m-1) (p+q)+p+1,\qquad l_{m,q,q}=m(p+q), \\& l_{v,1}=v(p+q)+1, \\& y_{n,m}^{\prime}=\sum_{i=l_{m,p,1}}^{l_{m,p,p}}Z_{n,i}(x),\qquad y_{n,m}^{\prime\prime }=\sum_{i=l_{m,q,1}}^{l_{m,q,q}}Z_{n,i}(x),\qquad y_{n,m}^{\prime\prime\prime }=\sum_{l=l_{v,1}}^{n}Z_{n,l}(x) \end{aligned}$$

and

$$S_{n}^{\prime}=\sum_{m=1}^{v}y_{n,m}^{\prime},\qquad S_{n}^{\prime\prime }=\sum_{m=1}^{v}y_{n,m}^{\prime\prime },\qquad S_{n}^{\prime\prime\prime }=y_{n,m}^{\prime\prime\prime }. $$

Then

3.5 $$ S_{n}=S_{n}^{\prime}+ S_{n}^{\prime\prime }+ S_{n}^{\prime\prime\prime }. $$

Next, some lemmas are given, which will be applied later.

### Lemma 3.1

Yang [3]

*Assume that* $\{X_{i}, i\geq1\}$ *is a sequence of* *ϕ*-*mixing random variables satisfying* $EX_{i}=0$, $E\vert X_{i}\vert ^{q}<\infty$ *for* $q\geq2$ *and* $i=1,2,\ldots $ *and* $\sum_{i=1}^{\infty}\phi^{1/2}(i)<\infty$. *Then there exists a positive constant* *C* *such that*

3.6 $$ E\Biggl\vert \sum_{i=1}^{n}X_{i} \Biggr\vert ^{q} \leq C \Biggl\{ \sum_{i=1} ^{n}E\vert X_{i}\vert ^{q}+ \Biggl( \sum _{i=1}^{n}EX_{i}^{2} \Biggr) ^{q/2} \Biggr\} $$

*for* $n\geq1$.

### Lemma 3.2

*Under the conditions of Theorem* 2.1, *we have*

3.7 $$ E\bigl(S_{n}^{\prime\prime }\bigr)^{2}\leq C \gamma_{1n},\qquad E\bigl(S_{n}^{\prime\prime\prime }\bigr)^{2} \leq C\gamma_{2n} $$

*and*

3.8 $$ P\bigl(\bigl\vert S_{n}^{\prime\prime }\bigr\vert \geq \gamma_{1n}^{1/3}\bigr)\leq C\gamma _{1n}^{1/3},\qquad P\bigl(\bigl\vert S_{n}^{\prime\prime\prime }\bigr\vert \geq\gamma _{2n}^{1/3}\bigr)\leq C\gamma_{2n}^{1/3}. $$

### Proof

By assumption (A1), it follows that $EZ_{n,i}^{2}=E\vert (nb_{n}\sigma_{n}(x))^{-1}\eta_{i}(x)\vert ^{2}\leq C\frac{1}{n^{2} b_{n} \sigma_{n}^{2} (x)}$. Hence, in terms of Lemma 3.1, we obtain that

3.9 $$\begin{aligned} E\bigl(S_{n}^{\prime\prime }\bigr)^{2} =&E \Biggl( \sum _{m=1}^{v}y_{n,m}^{\prime\prime } \Biggr) ^{2} \\ =&E \Biggl( \sum_{m=1}^{v} \sum_{i=l_{m,q,1}}^{l_{m,q,q}}Z _{n,i}(x) \Biggr) ^{2} \\ \leq& C \sum_{m=1}^{v}\sum _{i=l_{m,q,1}} ^{l_{m,q,q}}\frac{1}{n^{2} b_{n}\sigma_{n}^{2} (x)} \\ \leq& C\frac{vq}{n^{2} b_{n}\sigma_{n}^{2} (x)} \\ \leq& C\frac{n}{p+q} \frac{q}{n^{2} b_{n}\sigma_{n}^{2} (x)} \\ \leq& C\frac{q}{p+q}\frac{1}{nb_{n}\sigma_{n}^{2}(x)}=C \gamma_{1n} \end{aligned}$$

and that

3.10 $$\begin{aligned} E\bigl(S_{n}^{\prime\prime\prime }\bigr)^{2} =&E \bigl(y_{n,m}^{\prime\prime\prime }\bigr)^{2} =E \Biggl( \sum _{l=l _{v,1}}^{n}Z_{n,l}(x) \Biggr) ^{2} \\ \leq& C \sum_{l=l_{v,1}}^{n}\frac{1}{(nb_{n}^{1/2} \sigma_{n}(x))^{2}} \\ \leq& C\bigl[n-v(p+q)\bigr] \frac{1}{(nb_{n}^{1/2}\sigma_{n}(x))^{2}} \\ \leq& C\frac{p}{(nb_{n}^{1/2}\sigma_{n}(x))^{2}}=C\gamma _{2n}. \end{aligned}$$

Therefore, (3.7) holds. By Markov’s inequality and (3.9), (3.8) is obtained directly. The proof is complete. □

### Lemma 3.3

*For any integer* *k*, *there exist* $\zeta_{k_{s}} \in I_{k_{s}}$ ($s=0,1$) *such that*

3.11 $$ \bigl\vert \operatorname{Cov}(e_{i,k_{s}},e_{i+j,k_{s}})\bigr\vert \leq2 \bigl\{ \phi (j)\bigr\} ^{1/2}\bigl(f( \zeta_{k_{s}})\bigr)^{1/2}b_{n}^{1/2} $$

*and*

3.12 $$ \bigl\vert \operatorname{Cov}(e_{i,k_{s}-1},e_{i+j,k_{s}})\bigr\vert \leq2 \bigl\{ \phi (j)\bigr\} ^{1/2}\bigl(f( \zeta_{k_{s}})\bigr)^{1/2}b_{n}^{1/2}. $$

### Proof

By Theorem 5.1 in Roussas and Ioannides [9] and the proof of Corollary 2.1 in Carbon et al. [10], we can get the results directly. The details are omitted here. □

### Lemma 3.4

*Under the conditions of Theorem* 2.1, *we have*

3.13 $$ \vert s_{n}-1\vert \leq C\bigl\{ \gamma_{1n}^{1/2}+ \gamma _{2n}^{1/2}+u_{1}(q)\bigr\} , $$

*where* $s_{n}=\sqrt{\sum_{m=1}^{v}\operatorname{Var}(y_{n,m}^{\prime})}$.

### Proof

Set $\Gamma_{n}=\sum_{1\leq i< j\leq v} \operatorname{Cov}(y_{n,i}^{\prime},y_{n,j}^{\prime})$. Obviously,

3.14 $$ s_{n}^{2}= E\bigl(S_{n}^{\prime} \bigr)^{2}-2\Gamma_{n}. $$

Since $E(S_{n})^{2}=1$ and $E(S_{n}^{\prime })^{2}=E[S_{n}-(S_{n}^{\prime}+S _{n}^{\prime\prime })]^{2} =ES_{n}^{2}-2E[S_{n}(S_{n}^{\prime}+S_{n}^{\prime\prime })]+E(S_{n} ^{\prime}+S_{n}^{\prime\prime })^{2}$, we have

3.15 $$ \bigl\vert E\bigl(S_{n}^{\prime}\bigr)^{2}-1\bigr\vert =\bigl\vert E\bigl(S_{n}^{\prime }+S_{n}^{\prime\prime } \bigr)^{2} -2E\bigl[S _{n}\bigl(S_{n}^{\prime}+S_{n}^{\prime\prime } \bigr)\bigr]\bigr\vert \leq C\bigl(\gamma _{1n}^{1/2}+ \gamma_{2n} ^{1/2}\bigr). $$

On the other hand, by Lemma 3.3, it follows that

$$\begin{aligned} \vert \Gamma_{n}\vert \leq&\sum_{1\leq i< j\leq v} \bigl\vert \operatorname{Cov}\bigl(y_{n,i}^{\prime},y_{n,j}^{\prime} \bigr)\bigr\vert \\ \leq&\sum_{1\leq i< j\leq v}\sum_{s=l_{i,p,1}} ^{l_{i,p,p}}\sum_{t=l_{j,p,1}}^{l_{j,p,p}}\bigl\vert \operatorname{Cov}(Z_{n,s},Z_{n,t})\bigr\vert \\ \leq&\sum_{1\leq i< j\leq v}\sum_{s=l_{i,p,1}} ^{l_{i,p,p}}\sum_{t=l_{j,p,1}}^{l_{j,p,p}} \frac{1}{(nb_{n} \sigma_{n}(x))^{2}}\bigl\vert \operatorname{Cov}(\eta_{s}, \eta_{t})\bigr\vert \\ \leq&\sum_{1\leq i< j\leq v}\sum_{i=l_{i,p,1}} ^{l_{i,p,p}}\sum_{j=l_{j,p,1}}^{l_{j,p,p}} \frac{1}{(nb_{n} \sigma_{n}(x))^{2}} \biggl\vert \operatorname{Cov}\biggl( \biggl(\frac{1}{2}+k_{0}- \frac{x}{b _{n}}\biggr)e_{i,k_{0}}(x) \\ & {} +\biggl(\frac{1}{2}-k_{0}+ \frac {x}{b_{n}}\biggr)e_{i,k_{1}}(x), \biggl(\frac{1}{2}+k_{0}-\frac{x}{b_{n}} \biggr)e_{i,k _{0}}(x)+\biggl(\frac{1}{2}-k_{0}+ \frac{x}{b_{n}}\biggr)e_{i,k_{1}}(x) \biggr) \biggr\vert \\ \leq&\sum_{1\leq i< j\leq v}\sum_{s=l_{i,p,1}} ^{l_{i,p,p}}\sum_{t=l_{j,p,1}}^{l_{j,p,p}} \frac{1}{(nb_{n} \sigma_{n}(x))^{2}} \biggl\{ \biggl\vert \biggl(\frac{1}{2}+k_{0}- \frac{x}{b_{n}}\biggr)^{2} \operatorname{Cov}(e_{s,k_{0}},e_{t,k_{0}}) \biggr\vert \\ &{} +\biggl\vert \biggl(\frac{1}{2}+k_{0}- \frac{x}{b_{n}}\biggr) \biggl( \frac{1}{2}-k_{0}+ \frac{x}{b_{n}}\biggr) \operatorname{Cov}(e_{s,k_{0}},e_{t,k _{1}})\biggr\vert \\ & {} +\biggl\vert \biggl(\frac{1}{2}+k_{0}- \frac{x}{b_{n}}\biggr) \biggl( \frac{1}{2}-k_{0}+ \frac{x}{b_{n}}\biggr) \operatorname{Cov}(e_{s,k_{1}},e_{t,k _{0}})\biggr\vert \\ & {} +\biggl\vert \biggl(\frac{1}{2}-k_{0}+ \frac{x}{b_{n}}\biggr)^{2} \operatorname{Cov}(e_{s,k_{1}},e_{t,k_{1}}) \biggr\vert \biggr\} \\ \leq&\sum_{1\leq i< j\leq v}\sum_{s=l_{i,p,1}} ^{l_{i,p,p}}\sum_{t=l_{j,p,1}}^{l_{j,p,p}} \frac{4}{b_{n}^{3/2}(n \sigma_{n}(x))^{2}} \bigl\{ f^{1/2}(\zeta_{k_{0}})+3f^{1/2}( \zeta_{k _{1}}) \bigr\} \bigl\{ \phi\bigl(\vert s-t\vert \bigr)\bigr\} ^{1/2} \\ \leq &C\sum_{i=1}^{v-1}\sum _{j=i+1}^{v} \sum_{s=l_{i,p,1}}^{l_{i,p,p}} \sum_{t=l_{j,p,1}}^{l _{j,p,p}}\frac{1}{b_{n}^{3/2}(n\sigma_{n}(x))^{2}}\bigl\{ \phi \bigl(\vert s-t\vert \bigr)\bigr\} ^{1/2} \\ \leq& C\sum_{i=1}^{v-1} \sum _{s=l_{i,p,1}} ^{l_{i,p,p}}\frac{1}{b_{n}^{3/2}(n\sigma_{n}(x))^{2}}\sum _{m=q} ^{\infty}\bigl\{ \phi(m)\bigr\} ^{1/2} \\ \leq& C\sum_{i=1}^{v-1} \sum _{s=l_{i,p,1}} ^{l_{i,p,p}}\frac{1}{b_{n}^{3/2}(n\sigma_{n}(x))^{2}}\sum _{m=q} ^{\infty}m^{-\rho/2} \\ \leq& C\frac{q^{-\frac{1}{2}\rho+1}}{nb_{n} ^{3/2}(\sigma_{n}(x))^{2}}\\ \leq& Cu_{1}(q), \end{aligned}$$

which together with (3.14) and (3.15) yields (3.13). The proof is completed. □

Assume that $\{\eta_{nm}: m=1, \dots, v\}$ are independent random variables, and the distribution of $\eta_{nm}$ is the same as that of $y_{nm}^{\prime}$ for $m=1, \dots, v$. Let $T_{n}=\sum_{m=1}^{v}{\eta_{nm}}$, $B_{n}=\sum_{m=1}^{v}\operatorname{Var}( \eta_{n,m})$ and $\widetilde{F}_{n}(u)$, $G_{n}(u)$ and $\widetilde{G} _{n}(u)$ be the distribution functions of $S_{n}^{\prime}$, $T_{n}/\sqrt{B _{n}}$ and $T_{n}$, respectively. Clearly,

3.16 $$ B_{n}=s_{n}^{2}, \qquad \widetilde{G}_{n}(u)=G_{n}(u/s_{n}). $$

### Lemma 3.5

*Under the conditions of Theorem* 2.1, *we have*

3.17 $$ \sup_{u}\bigl\vert G_{n}(u)-\Phi(u)\bigr\vert \leq C\gamma_{3n}. $$

### Proof

By Lemma 3.1, $\vert z_{n,i}\vert \leq\frac{e_{i,k_{0}}(x)+e _{i,k_{1}}(x)}{nb_{n}\sigma_{n}(x)}$ and assumption (A1), we have

$$\begin{aligned}& \sum_{m=1}^{v}E\vert \eta_{n,m}\vert ^{2+\delta} \\& \quad \leq C \Biggl\{ \sum_{m=1}^{v}\sum _{i=l_{m,p,1}}^{l_{m,p,p}}E\bigl\vert Z_{n,i}(x) \bigr\vert ^{2+\delta}+ \sum_{m=1}^{v} \Biggl( \sum_{i=l_{m,p,1}}^{l_{m,p,p}} EZ_{n,i}^{2}(x) \Biggr) ^{1+\delta/2} \Biggr\} \\& \quad \leq C \biggl\{ pvb_{n} \biggl( \frac{1}{nb_{n}\sigma_{n}(x)} \biggr) ^{2+ \delta} +v \biggl[ \frac{pb_{n}}{(nb_{n}\sigma_{n}(x))^{2}} \biggr] ^{1+ \delta/2} \biggr\} \\& \quad \leq Cvb_{n} \biggl[ \frac{p}{(nb_{n}\sigma_{n}(x))^{2}} \biggr] ^{1+ \delta/2} \\& \quad \leq C\frac{p^{1+\delta/2}b_{n}}{p+q} \biggl( \frac{1}{n^{1+\delta}(b _{n}\sigma_{n}(x))^{2+\delta}} \biggr) \\& \quad \leq C\frac{p^{\delta/2}}{(nb_{n})^{1+\delta}(\sigma_{n}(x))^{2+ \delta}}=C\gamma_{3n}, \end{aligned}$$

which together with $B_{n}=s_{n}^{2}\rightarrow1$ yielded by Lemma 3.4 implies that (3.17) holds by the Berry-Esséen theorem. □

### Lemma 3.6

*Let* $\{X_{i}, i\geq1\}$ *be a sequence of* *ϕ*-*mixing random variables*, *and let* $\eta_{l}=\sum_{i=(l-1)(p+q)+1}^{(l-1)(p+q)+p}X_{i}$, *where* $1\leq l\leq k$. *If* $\frac{1}{r}+\frac{1}{s}=1$, *where* $r>0$, $s>0$, *then*

3.18 $$ \Biggl\vert E\exp \Biggl( it\sum_{l=1}^{v} \eta_{l} \Biggr) -\prod_{l=1}^{v}E \exp(it\eta_{l})\Biggr\vert \leq \vert t\vert \phi^{1/s}(q) \sum_{i=1}^{v}\Vert \eta_{l}\Vert _{r}. $$

### Proof

Obviously,

3.19 $$\begin{aligned}& \Biggl\vert E\exp \Biggl( it\sum_{l=1}^{v} \eta_{l} \Biggr) -\prod_{l=1}^{v}E \exp(it\eta_{l})\Biggr\vert \\& \quad \leq\Biggl\vert E\exp \Biggl( it\sum_{l=1}^{v} \eta_{l} \Biggr) -E \exp \Biggl( it\sum_{l=1}^{v-1} \eta_{l} \Biggr) E\exp(it\eta_{v})\Biggr\vert \\& \qquad {} +\Biggl\vert E\exp \Biggl( it\sum_{l=1}^{v-1} \eta_{l} \Biggr) E\exp(it \eta_{v})-\prod _{l=1}^{v}E\exp(it\eta_{l})\Biggr\vert \\& \quad =:I_{1}+I_{2}. \end{aligned}$$

Noting that $e^{ix}=\cos x+i\sin x$, $\sin(x+y)=\sin x\cos y+\cos x \sin y$ and $\cos(x+y)=\cos x\cos y-\sin x\sin y$, we get

3.20 $$\begin{aligned} I_{1} =&\Biggl\vert E\exp \Biggl( it\sum _{l=1}^{v}\eta_{l} \Biggr) -E \exp \Biggl( it\sum_{l=1}^{v-1} \eta_{l} \Biggr) E\exp(it\eta_{v})\Biggr\vert \\ \leq&\Biggl\vert \operatorname{Cov}\Biggl( \cos \Biggl( t\sum _{l=1}^{v-1}\eta _{l} \Biggr), \cos(t \eta_{v}) \Biggr) \Biggr\vert +\Biggl\vert \operatorname{Cov}\Biggl( \sin \Biggl( t \sum_{l=1}^{v-1} \eta_{l} \Biggr), \sin(t\eta_{v}) \Biggr) \Biggr\vert \\ &{} + \Biggl\vert \operatorname{Cov}\Biggl( \sin \Biggl( t\sum _{l=1}^{v-1}\eta _{l} \Biggr), \cos(t \eta_{v}) \Biggr) \Biggr\vert +\Biggl\vert \operatorname{Cov}\Biggl( \cos \Biggl( t \sum_{l=1}^{v-1} \eta_{l} \Biggr), \sin(t\eta_{v}) \Biggr) \Biggr\vert \\ =:&I_{11}+I_{12}+I_{13}+I_{14}. \end{aligned}$$

From Theorem 5.1 in Roussas and Ioannides [9] and $\vert \sin x\vert \leq \vert x\vert $, it follows that

3.21 $$ I_{12}\leq C\phi^{1/s}(q)\bigl\Vert \sin(t \eta_{v})\bigr\Vert _{r}\leq C\vert t\vert \phi^{1/s}(q) \Vert \eta_{v}\Vert _{r},\qquad I_{14}\leq C\vert t\vert \phi ^{1/s}(q)\Vert \eta_{v}\Vert _{r}. $$

Also, by $\cos(2x)=1-2\sin^{2}x$, we get that

3.22 $$\begin{aligned} I_{11} =&\Biggl\vert \operatorname{Cov}\Biggl( \cos \Biggl( t\sum _{l=1}^{v-1}\eta _{l} \Biggr), 1-2\sin^{2}(t\eta_{v}/2) \Biggr) \Biggr\vert \\ =&2\Biggl\vert \operatorname{Cov}\Biggl( \cos \Biggl( t\sum _{l=1}^{v-1} \eta_{l} \Biggr), \sin^{2}(t\eta_{v}/2) \Biggr) \Biggr\vert \\ \leq& C\phi^{1/s}(q)E^{1/r}\bigl\vert \sin(t \eta_{v}/2)\bigr\vert ^{2r} \\ \leq& C\phi^{1/s}(q)E^{1/r} \bigl\vert \sin(t\eta_{v}/2)\bigr\vert ^{r} \\ \leq& C\phi^{1/s}(q)\Vert \eta_{v}\Vert _{r}. \end{aligned}$$

Similarly,

3.23 $$ I_{13}\leq C\phi^{1/s}(q)\Vert \eta_{v} \Vert _{r}. $$

A combination of (3.19)-(3.23) yields that

$$\Biggl\vert E\exp \Biggl( it\sum_{l=1}^{v} \eta_{l} \Biggr) -\prod_{l=1}^{v}E \exp(it\eta_{l})\Biggr\vert \leq C\phi^{1/s}(q)\Vert \eta _{v}\Vert _{r}+I_{2}. $$

Repeating the procedure above makes (3.18) hold. The proof is completed. □

### Lemma 3.7

*Under the conditions of Theorem* 2.1, *we have*

3.24 $$ \sup_{u}\bigl\vert \widetilde{F}_{n}(u)- \widetilde{G}_{n}(u)\bigr\vert \leq C\bigl\{ \gamma_{3n}+u_{2}^{1/2}(q) \bigr\} . $$

### Proof

Let $\varphi(t)$ and $\psi(t)$ be the characteristic functions of $S_{n}^{\prime}$ and $T_{n}$, respectively. Noting

$$\psi(t)=E\bigl(\exp\{itT_{n}\}\bigr)=\prod _{m=1}^{v}E\exp(it\eta_{n,m}) =\prod _{m=1}^{v}E\exp\bigl(ity_{n,m}^{\prime} \bigr), $$

we have

3.25 $$ \bigl\vert \varphi(t)-\psi(t)\bigr\vert =\Biggl\vert E\exp \Biggl( it\sum _{l=1}^{v}\eta _{l} \Biggr) - \prod_{l=1}^{v} E\exp(it \eta_{l})\Biggr\vert \leq \vert t\vert \phi^{1/2}(q)\sum _{i=1}^{v}\bigl\Vert y_{n,m}^{\prime} \bigr\Vert _{2}. $$

Also, from Lemma 3.1, it follows that

$$E\bigl(y_{n,m}^{\prime}\bigr)^{2}=E \Biggl( \sum _{i=l_{m,p,1}}^{l_{m,p,p}}Z_{n,i}(x) \Biggr) ^{2} \leq C \sum_{i=l_{m,p,1}}^{l_{m,p,p}} \frac{1}{(nb_{n}^{1/2} \sigma_{n}(x))^{2}} \leq C\frac{p}{(nb_{n}^{1/2}\sigma_{n}(x))^{2}}. $$

Then we have

$$\begin{aligned} \bigl\vert \varphi(t)-\psi(t)\bigr\vert \leq&\vert t\vert \phi ^{1/2}(q)\sum_{i=1}^{v} \biggl[ \frac{p}{(nb_{n}^{1/2}\sigma_{n}(x))^{2}} \biggr] ^{1/2} \\ \leq&\vert t\vert q^{-\rho/2}\frac{n}{p+q} \biggl[ \frac {p}{(nb_{n}^{1/2}\sigma _{n}(x))^{2}} \biggr] ^{1/2} \\ \leq& C\vert t\vert \bigl( q^{\rho}p b_{n} \sigma_{n}^{2}(x) \bigr) ^{-1/2}=C\vert t\vert u_{2}(q), \end{aligned}$$

which implies that

3.26 $$ \int_{-T}^{T}\biggl\vert \frac{\varphi(t)-\psi(t)}{t}\biggr\vert \,dt\leq Cu_{2}(q)T. $$

On the other hand, by $\widetilde{G}_{n}(u)=G_{n}(u/s_{n})$ and Lemma 3.5, we get

$$\begin{aligned}& \sup_{u}\bigl\vert \widetilde{G}_{n}(u+y)- \widetilde{G}_{n}(u)\bigr\vert \\& \quad = \sup_{u}\bigl\vert G_{n} \bigl((u+y)/s_{n}\bigr)-G_{n}(u/s_{n})\bigr\vert \\& \quad \leq\sup_{u}\bigl\vert G_{n} \bigl((u+y)/s_{n}\bigr)-\Phi\bigl((u+y)/s_{n}\bigr)\bigr\vert \\& \qquad {} +\sup_{u}\bigl\vert \Phi\bigl((u+y)/s_{n} \bigr)-\Phi(u/s_{n})\bigr\vert +\sup_{u}\bigl\vert G_{n}(u/s_{n})-\Phi(u/s_{n})\bigr\vert \\& \quad \leq2\sup_{u}\bigl\vert G_{n}(u)-\Phi(u)\bigr\vert +\sup_{u}\bigl\vert \Phi\bigl((u+y)/s_{n} \bigr)-\Phi(u/s_{n})\bigr\vert \\& \quad \leq C\bigl\{ \gamma_{3n}+\vert y\vert /s_{n}\bigr\} \leq C\bigl\{ \gamma _{3n}+\vert y\vert \bigr\} . \end{aligned}$$

Therefore,

$$T\sup_{u} \int_{\vert y\vert \leq c/T}\bigl\vert \widetilde {G}_{n}(u+y)- \widetilde{G}_{n}(u)\bigr\vert \,dy\leq CT \int_{\vert y\vert \leq c/T}\bigl\{ \gamma_{3n}+\vert y\vert \bigr\} \,dy \leq C\{\gamma_{3n}+1/T\}, $$

which together with (3.26) implies

$$\begin{aligned} \sup_{u}\bigl\vert \widetilde{F}_{n}(u)- \widetilde{G}_{n}(u)\bigr\vert \leq& \int _{-T}^{T}\biggl\vert \frac{\varphi(t)-\psi(t)}{t} \biggr\vert \,dt+T\sup_{u} \int_{\vert y\vert \leq c/T}\bigl\vert \widetilde {G}_{n}(u+y)- \widetilde{G}_{n}(u)\bigr\vert \,dy \\ \leq& C\bigl\{ \gamma_{3n}+u_{2}(q)T+1/T\bigr\} =C\bigl\{ \gamma_{3n}+u _{2}^{1/2}(q)\bigr\} \end{aligned}$$

by the Esséen theorem and letting $T=u_{2}^{-1/2}(q)$. The proof is complete. □

### Lemma 3.8

Yang [11]

*Suppose that* $\{ \zeta_{n}:n \geq1 \} $ *and* $\{ \eta_{n}:n\geq1 \} $ *are two random variable sequences*, $\{ \gamma_{n}:n\geq1 \} $ *is a positive constant sequence and* $\gamma_{n}\to0$. *If*

$$\sup_{u}\bigl\vert F_{\zeta_{n}}(u)-\Phi(u)\bigr\vert \leq C\gamma_{n}, $$

*then for any* $\varepsilon> 0$,

3.27 $$ \sup_{u}\bigl\vert F_{\zeta_{n}+\eta_{n}}(u)-\Phi(u)\bigr\vert \leq C\bigl\{ \gamma _{n}+ \varepsilon+ P\bigl(\vert \eta_{n}\vert \geq\varepsilon\bigr) \bigr\} . $$

In what follows, we can give the proof of Theorem 2.1.

### Proof of Theorem 2.1

It is easy to see that

$$\begin{aligned}& \sup_{u}\bigl\vert \widetilde{F}_{n}(u)-\Phi(u) \bigr\vert \\& \quad \leq\sup_{u}\bigl\vert \widetilde{F}_{n}(u)- \widetilde{G}_{n}(u)\bigr\vert + \sup_{u}\bigl\vert \widetilde{G}_{n}(u)-\Phi(u/\sqrt{B_{n}})\bigr\vert + \sup_{u}\bigl\vert \Phi(u/\sqrt{B_{n}})-\Phi(u) \bigr\vert \\& \quad =:J_{1n}+J_{2n}+J_{3n}. \end{aligned}$$

By Lemmas 3.7, 3.5 and 3.4, we can obtain

$$\begin{aligned}& J_{1n}=\sup_{u}\bigl\vert \widetilde{F}_{n}(u)- \widetilde{G}_{n}(u)\bigr\vert \leq C\bigl\{ \gamma_{3n}+u_{2}^{1/2}(q) \bigr\} , \\& J_{2n}=\sup_{u}\bigl\vert G_{n}(u/ \sqrt{B_{n}})-\Phi(u/\sqrt {B_{n}})\bigr\vert = \sup _{u}\bigl\vert G_{n}(u)-\Phi(u)\bigr\vert \leq C \gamma_{3n} \end{aligned}$$

and

$$J_{3n}=\sup_{u}\bigl\vert \Phi(u/ \sqrt{B_{n}})-\Phi(u)\bigr\vert \leq \vert s_{n}-1\vert \leq C\bigl\{ \gamma_{1n}^{1/2}+\gamma_{2n}^{1/2}+u_{1}(q) \bigr\} , $$

which together with (3.5), (3.8) and (3.27) implies (2.1). The proof is completed. □
